# Special Issue: Zinc Oxide Nanostructures: Synthesis and Characterization

**DOI:** 10.3390/ma11060873

**Published:** 2018-05-23

**Authors:** Sotirios Baskoutas

**Affiliations:** Department of Materials Science, University of Patras, 26500 Patras, Greece; bask@upatras.gr; Tel.: +30-261-096-9349

**Keywords:** ZnO, synthesis, characterization, nanoparticles, nanorods, quantum wires, thin films

## Abstract

Zinc oxide (ZnO) is a wide band gap semiconductor with an energy gap of 3.37 eV at room temperature. It has been used considerably for its catalytic, electrical, optoelectronic, and photochemical properties. ZnO nanomaterials, such as quantum dots, nanorods, and nanowires, have been intensively investigated for their important properties. Many methods have been described in the literature for the production of ZnO nanostructures, such as laser ablation, hydrothermal methods, electrochemical deposition, sol–gel methods, Chemical Vapour Deposition, molecular beam epitaxy, the common thermal evaporation method, and the soft chemical solution method. The present Special Issue is devoted to the Synthesis and Characterization of ZnO nanostructures with novel technological applications.

Among various metal oxide materials, ZnO presents itself as a multifunctional material due to its own properties and functionalities. The properties of ZnO include its wide band gap (3.37 eV), high exciton binding energy (60 meV) [1,2], biocompatibility, and ease of fabrication. Due to its excellent properties, ZnO is widely used for various potential applications, such as catalysis, solar cells, ultraviolet (UV) lasers, light-emitting diodes, photo-detectors, sensors (chemical, bio-, and gas), and optical and electrical devices [3]. Among various applications, the use of ZnO nanomaterials as a photocatalyst has attracted particular interest due to their large surface area; wide band gap; ease of fabrication and cost effective synthesis; and biocompatible and environmentally benign nature [4].

The synthesis of large-scale arrayed one-dimensional (1D) ZnO nanostructures, including nanowires, nanorods, nanobelts, and whiskers, is an important step for the fabrication of functional nano/microdevices. Recently, because of its high-temperature strength and rigidity, as well as excellent chemical stability, small-diameter ZnO whiskers have received great attention for industrial applications as reinforcement phase in composite materials. ZnO whiskers with a high aspect ratio have also been successfully used as a probing tip to develop new precise high-resolution imaging techniques for atomic force microscopy and scanning tunneling microscopy.

This Special Issue covers 3 review articles, 1 brief report, and 13 research articles. First of all, Chaudhary et al. [5] present an overview of the current advancements of ZnO-nanomaterial-based chemical sensors. Various operational factors, such as the effect of size, morphologies, compositions, and their respective working mechanisms, along with the selectivity, sensitivity, detection limit, and stability are discussed in this article. Scherzad et al. [6] in their review article summarize the existing data regarding the DNA damage that ZnO nanoparticles (NPs) induce, and focus on the possible molecular mechanisms underlying genotoxic events. Wang et al. [7] present a review on three-dimensional (3D) ZnO hierarchical nanostructures, and summarize major advances in solution phase synthesis and applications in the environment and electrical/electrochemical devices. They present the principles and growth mechanisms of ZnO nanostructures via different solution methods with an emphasis on rational control of the morphology and assembly. Then, they discuss the applications of 3D ZnO hierarchical nanostructures in photocatalysis, field emissions, electrochemical sensors, and lithium ion batteries. In the research articles, Giannouli et al. [8] present a comparative assessment of nanowire- versus nanoparticle-based ZnO dye-sensitized solar cells (DSSCs) in order to investigate the main parameters that affect device performance. Bittner at al. [9] perform a low-temperature fabrication of flexible ZnO photo-anodes for dye-sensitized solar cells (DSSCs) by templated electrochemical deposition of films in an enlarged and technically simplified deposition setup to demonstrate the feasibility of the scale up of the deposition process. Kwoka et al. [10] present the results of detailed X-ray photoelectron spectroscopy (XPS) studies combined with atomic force microscopy (AFM) investigation concerning the local surface chemistry and morphology of nanostructured ZnO thin films. Giuli et al. [11] report on the structural and electrochemical characterization of Fe-doped ZnO samples with varying dopant concentrations, which may potentially serve as anodes for rechargeable lithium-ion batteries (LIBs). Xia et al. [12] present two new functional materials based on zinc oxide (ZnO)—a legacy material in semiconductors but exceptionally novel to solid state ionics—that are developed as membranes in solid oxide fuel cells (SOFCs) for the first time. Sarwar et al. [13] in their work study an NH_4_OH treatment to provide an optimum morphological trade-off to Ga-doped ZnO nanorods (n-GZR)/p-Si heterostructure characteristics. Quiñones et al. [14] in their paper use perfluorinated phosphonic acid modifications to modify zinc oxide (ZnO) nanoparticles because they create a more stable surface due to the electronegativity of the perfluoro head group. Umar et al. [15] report the growth of In-doped ZnO (IZO) nanomaterials, i.e., stepped hexagonal nanorods and nanodisks, by a thermal evaporation process using metallic zinc and indium powders in the presence of oxygen. The as-grown IZO nanomaterials were investigated by several techniques in order to examine their morphological, structural, compositional, and optical properties. From an application point of view, the grown IZO nanomaterials were used as a potential scaffold to fabricate sensitive phenyl hydrazine chemical sensors based on the I–V technique. In Pimentel et al.’s work [16], tracing and Whatman papers were used as substrates to grow zinc oxide (ZnO) nanostructures. The time-resolved photocurrent of the devices in response to UV being turned on/off was investigated and it has been observed that the ZnO nanorod arrays grown on the Whatman paper substrate present a responsivity that is 3 times greater than the ones grown on tracing paper. By using ZnO nanorods, the surface area-to-volume ratio will increase and will improve the sensor’s sensibility, making these types of materials good candidates for low-cost and disposable UV sensors. Sagasti et al. [17] synthesized a nanostructured ZnO layer onto a Metglas magnetoelastic ribbon to immobilize hemoglobin (Hb) on it and study the Hb’s electrochemical behavior towards hydrogen peroxide. Zhou et al. [18] fabricated a highly sensitive acetone chemical sensor using a ZnO nanoballs-modified silver electrode. Ibrahim et al. [19] reported a facile synthesis, characterization, and electrochemical-sensing application of ZnO nanopeanuts synthesized by a simple aqueous solution process and characterized by various techniques in order to confirm the compositional, morphological, structural, crystalline phase, and optical properties of the synthesized material. Beshkar et al. [20] have demonstrated a facile formation of CuO nanostructures on copper substrates by the oxidation of copper foil in ethylene glycol at 80 °C. The hydrophobic property of the products was characterized by means of water contact angle measurement. After simple surface modification with stearic acid and polydimethylsiloxane (PDMS), the resulting films showed hydrophobic and even superhydrophobic characteristics due to their special surface energy and nano-microstructure morphology. Finally, in their brief report, Hoffman et al. [21] use two-dimensional fluorescence difference spectroscopy (2-D FDS) to determine the unique spectral signatures of zinc oxide (ZnO), magnesium oxide (MgO), and a 5% magnesium zinc oxide nanocomposite (5% Mg/ZnO), which was then used to demonstrate the change in spectral signature that occurs when physiologically important proteins, such as angiotensin-converting enzyme (ACE) and ribonuclease A (RNase A), interact with ZnO nanoparticles (NPs).
